# Adopting a Societal Perspective in Health-Economic Evaluation: Analysis of Nine HTA Methodological Guidelines on How to Integrate Societal Costs

**DOI:** 10.3390/jmahp14010010

**Published:** 2026-02-10

**Authors:** Chloé Gervès-Pinquié, Hortense Nanoux, Sarah Akarkoub, Rémi Brazeilles, Henri Bonnabau, Katell Le Lay

**Affiliations:** 1 HEOR Department, IQVIA, 92060 La Defense, France; 2 HEOR Department, Roche, 92100 Boulogne Billancourt, France; 3 Real World Data Department, IQVIA, 92060 La Defense, France

**Keywords:** societal perspective, societal costs, health technology assessment, guidelines, multiple factor analysis

## Abstract

Objective: The perspective applied in Health Technology Assessment (HTA) has recently been presented as a central methodological debate in HTA. Beyond expected effects on patients’ health and quality of life, health technologies can affect broader societal domains, such as the labor market and informal care. Ensuring comparability between treatments when estimating their impact on society relies strongly on transparency regarding the methodology used to measure and value societal costs. This study aims to describe and summarize current recommendations of HTA bodies on adopting societal perspectives in HTA. Methods: A scoping review of HTA guidelines was conducted, and findings were validated through semi-structured interviews with academic and industry HTA experts. Guidelines from nine countries (Australia, Canada, France, Germany, The Netherlands, Spain, Sweden, United Kingdom, United States of America) were analyzed using an extraction grid covering four domains: out-of-pocket/copayment costs, informal care costs, productivity losses, and unrelated health care costs. Exploratory analyses (multiple factor analysis and clustering) assessed whether recommendations for a societal perspective as a base case were homogeneous across countries. Results: A societal perspective was recommended as a base case in 56% of the guidelines. Marked cross-country heterogeneity was observed for measurement methods. Only The Netherlands recommended a specific instrument for both informal care and productivity losses (iPCQ). The most frequently cited valuation approaches were the opportunity cost method (informal care) and the friction cost method (productivity losses). Three clusters of countries were identified: The Netherlands/Canada (1); US/Sweden/UK/Germany (2); and Spain/France/Australia (3). Exploratory analyses indicated limited alignment between endorsing the inclusion of societal costs and recommending the societal perspective as base case. Conclusions: This preliminary work highlights the need for explicit methodological guidance on societal cost estimation within HTA. HTA bodies could draw on the Netherland’s guidelines and cost database and develop a national “societal perspective doctrine”—including core cost sets, standardized measurement tools (e.g., iPCQ; diary methods), and structured involvement of patients and caregivers—to enhance comparability and decision relevance.

## 1. Background

In a context of scarce resources and unlimited healthcare needs, health economic evaluation plays a central role in informing decision-making regarding healthcare interventions [1,2,3], with critical implications for reimbursement and pricing policies [1,4,5,6,7]. It is increasingly acknowledged that healthcare interventions may generate, in addition to their direct use of healthcare resources and their effects on patients’ health, broader impacts on various social dimensions. These include changes in labor productivity, time devoted to informal (non-professional) care, effects on the health and well-being of other individuals, and even environmental consequences such as carbon footprint [2,6,8].

The choice of the perspective from which the results of a health economic evaluation are considered has recently been described by the *impact HTA* task force as “one of the areas of greatest methodological discussion” [9]. Challenges and opportunities related to the integration of societal costs into health economic assessments have also been prominently discussed at recent ISPOR Europe conferences and in the growing body of scientific literature on this topic [10,11,12,13].

The growing attention paid to this topic parallels demographic trends, notably population ageing and the increasing prevalence of age-related diseases, as well as the heightened visibility of family and informal care in certain rare and chronic conditions. It is also closely connected to the broader shift toward patient-centered care, which emphasizes the impact of diseases and treatments on patients’ ability to work or return to work, increasingly captured through Patient-Reported Measures (PRMs). The substantial body of literature estimating the societal costs of dementia, multiple sclerosis, and several rare diseases such as spinal muscular atrophy [14,15,16,17,18]—conditions typically associated with extensive informal caregiving demands or major productivity losses for patients and carers—illustrates that some therapeutic areas are particularly relevant for adopting a societal perspective in health technology assessment (HTA) [19,20,21,22,23].

While some HTA bodies, such as the United Kingdom’s National Institute for Health and Care Excellence (NICE) or Canada’s Drug and Health Technology Agency (CADTH), continue to prioritize the healthcare payer perspective as the reference analysis in health economic evaluation, others, like The Netherlands National Health Institute (NHI) considers this could lead to sub-optimal resource allocation and biased health policies. Some HTA bodies, like the French Haute Autorité de Santé (HAS), adopted an intermediate position, in which the societal perspective is recommended only as a complementary analysis [24]. According to the *impact HTA* review, more than 41% of the HTA guidelines recommended the use of a societal perspective—either as base case or co-base case—in 2019 [9]. Despite these recommendations, the practical integration of societal costs into economic evaluations remains limited. Pennington’s review of NICE technology appraisals up to 2020 showed that only 3% of evaluations incorporated caregiver health-related quality of life (HRQoL) into cost–utility analyses (CUAs), even though NICE guidance advocates including such spillover effects in the base-case analysis [25]. In response to these persistent gaps, the *Spillovers in Health Economic Evaluation and Research* (SHEER) task force convened to establish a “consensus of academic opinion on appropriate methods” for incorporating spillovers in economic models [10]. Further insight into HTA practice was provided by Breslau et al., who showed that very few HTA guidelines formally recommend the inclusion of societal or novel value elements in base-case analyses. Nonetheless, their work highlights a clear increase in the proportion of guidelines integrating such value elements in more recent publications [26].

Under the healthcare or health-payer perspective, the decision-maker seeks to maximize health outcomes within a fixed budget. In contrast, adopting a societal perspective requires maximizing social welfare while accounting for a broader set of resources and costs [5,27]. Adopting a narrower perspective, such as the health care payer’s, may therefore lead to different conclusions and decisions than those derived from a broader societal perspective, which includes all costs and effects related to the intervention, regardless of who incurs them [14]. A recent review demonstrated that the probability of an intervention being deemed cost-effective was consistently higher when analyses were conducted from a societal perspective compared with a health-system perspective [28].

There are currently no clear methodological standards or consensus—either among researchers or HTA bodies—regarding the identification, measurement and valuation of the resources that should be included under a societal perspective. This lack of harmonization makes the integration of societal costs into health economic evaluations considerably more complex than the inclusion of direct medical costs [9]. The unresolved methodological debates surrounding how these societal costs should be measured and valued inherently increase the uncertainty associated with cost-effectiveness results when societal costs are incorporated into economic models. This can be perceived as a disincentive by manufacturers, particularly given that the inclusion of societal costs often requires additional, specific, and time-consuming data collection efforts [29,30].

Developing clear methodological guidelines on the inclusion and estimation of societal costs remains a considerable challenge for HTA bodies, regardless of whether such costs are recommended for base-case cost-effectiveness analyses or only as complementary analyses. Establishing transparent and standardized methods is essential both to ensure comparability across interventions and to make the adoption of a societal perspective feasible for manufacturers within health economic evaluations. Therefore, this article aimed to examine HTA bodies’ recommendations regarding the application of a societal perspective in health economic assessment, based on their most recently published guidelines. The first objective was to describe and synthesize current recommendations on the adoption of a societal cost perspective. The second objective was to identify potential clusters of countries according to their positions on the societal perspective.

## 2. Materials and Methods

The methodological approach adopted for this work comprised four stages. To address the first objective—describing and synthesizing current HTA bodies’ recommendations on adopting a societal perspective—we selected the relevant HTA guidelines and developed both a glossary of societal cost categories and an extraction grid for their analysis. This initial framework was validated by academic experts, both with respect to the countries considered relevant for inclusion and the appropriateness of the societal cost categories retained in the extraction grid (Stage 1). The extraction of guideline content was then performed and validated by HTA country experts from industry (Stage 2). A descriptive analysis of the resulting database—validated from both methodological and empirical standpoints—was conducted thereafter (Stage 3). To achieve the second objective, we performed exploratory analyses using multiple factor analysis (MFA) and cluster analysis to identify potential groups of countries based on their recommendations regarding the societal perspective (Stage 4).

### 2.1. Stage 1. Data Source, Glossary and Academic Validation

The first stage of the analysis consisted in the selection of HTA guidelines from countries that publish most extensively on societal costs, written in English, Spanish or French, and representing major pharmaceutical markets with established HTA systems (Table 1). Country selection was informed by the findings of a preliminary literature review conducted upstream to help scope the project [28], which helped identify countries where societal costs have been particularly studied. Additional selection criteria included the availability of published guidelines in the relevant languages that remain in use as reference documents in the respective countries, the expertise of the academic experts involved in the study—who contributed to narrowing down the list to those countries deemed most relevant—and the feasibility of engaging local experts from Roche and IQVIA to provide country-specific insights on societal cost considerations. A targeted literature search was conducted on grey literature and the HTA bodies’ websites to identify HTA guidelines or doctrines, local costing guidelines, and any guidelines specifically addressing societal costs across different countries.

Societal costs encompass all relevant costs directly and indirectly related to a condition and/or intervention regardless of who bears them (e.g., national health insurance, complementary health insurance, patient/caregiver, employers). They include both medical and non-medical costs arising within and outside the healthcare sector—for example, in the education, justice, labor, or environmental sectors [9,31]. The most recent HTA guidelines available for each selected country were included, spanning publications from 2003 to 2022.

To extract the relevant information from the selected guidelines, we developed an Excel-based extraction grid that captured the following variables: country, year of publication, recommendation regarding the societal perspective (base case or complementary analysis only), and a detailed inventory of items considered as societal costs. These items included out-of-pocket and copayment costs, informal care costs, productivity losses (distinguishing leisure time, unpaid work, anticipated retirement, presenteeism, and absenteeism), education-related costs, environmental costs, and unrelated healthcare costs, as well as the measurement and valuation methods recommended for each cost category. The list of extracted societal cost items corresponds to both the informal healthcare sector and non-healthcare sector components defined in the ISPOR Second Panel’s impact inventory template [32].

Macro-economic variables from the Organization for Economic Co-operation and Development, the World Health Organization and the International Monetary Fund [33,34,35] were added to the database to contextualize HTA bodies’ recommendations regarding the societal perspective.

In order to facilitate understanding of the concepts used in this study surrounding societal costs, a glossary was developed (Table S1—Supplementary Materials). It was primarily based on the definitions found in the HTA guidelines, complemented by terminology identified through a recent systematic review on the topic [28]. This glossary was shared with academic experts, prior to the presentation of the extraction grid, to ensure common alignment on the concepts and categories to be extracted from the recommendations. The glossary was subsequently revised according to the feedback gathered during the experts’ interviews.

Academic experts, selected for their expertise in health economics and HTA methodology, were also provided with a pre-read PowerPoint presentation detailing the extraction grid developed to collect HTA bodies’ recommendations on societal perspectives. A one-hour semi-structured interview was subsequently conducted in March 2023 in the form of a joint round-table discussion. A meeting summary was circulated and validated to resolve any points of disagreement between experts. The extraction grid was revised accordingly, following the experts’ recommendations. Potential conflicts of interest were disclosed in the dedicated section of the manuscript.

### 2.2. Stage 2. Data Extraction and Validation with HTA Country Experts

To minimize methodological bias and errors (e.g., misinterpretation of definitions for societal cost categories), the data-extraction process followed a double-blinded peer review approach with two independent researchers extracting the data in parallel. In cases of disagreement, the researchers first sought to reach consensus through discussion. If consensus could not be achieved, a third researcher acted as an adjudicator, reviewing the disputed items and applying predefined rules (e.g., prioritizing explicit definitions provided in the guideline and adhering to standard HTA terminology). The adjudicator’s decision was considered final.

To validate our interpretation of the country-specific HTA guidelines, the data extraction was reviewed by local expert volunteers from IQVIA or Roche affiliates between May and September 2023. These country experts were volunteers from IQVIA or Roche affiliates with experience in national HTA processes and in submissions including societal costs.

The glossary and the preliminary completed extraction grid (in PowerPoint format) were sent to the HTA country experts prior to their interview. Experts could participate either through a semi-structured two-hour interview or by completing the interview in questionnaire form (submitted online or discussed orally if clarification was needed). Country experts worked independently and received questionnaires tailored to the HTA recommendations applicable in their country, in addition to a set of questions common across countries (Table S2).

For semi-structured interviews, the interview guide was shared in advance to facilitate discussion. The guide consisted of multiple-choice and closed-ended questions to ensure reproducibility (Table S2—Supplementary Materials). The selected topics were as follows:

- Contextual questions (3 questions): overview of the number of HTA bodies, their scope, funding structure, and the binding nature of their recommendations.
- General recommendations on the societal perspective (8 questions): covering assessment validation and additional information requirements, including whether the societal perspective is recommended as a base case or complementary analysis, and how societal costs are defined.
- Specific methodological requirements, divided into 4 categories (24 questions): direct non-medical costs and out-of-pocket/copayment costs, informal care costs, productivity losses and unrelated health care costs. Questions addressed definitions, measurement and valuation methods, and expected data sources.

When country experts agreed with the study team’s interpretation of the guidelines, no modifications were made to the extraction grid. All expert responses were documented and confirmed through written reports to ensure accurate and consistent interpretation.

### 2.3. Stage 3. Data Analysis

The extracted data were recoded to enable descriptive analyses, expressed as frequencies either by country or across all countries. All the variables have been binarized to obtain harmonized “yes/no” outputs for each parameter in the extraction grid, regardless of the level of detail available in the guidelines [36].

### 2.4. Stage 4. Exploratory Analysis

Exploratory analyses were conducted to refine the interpretation of the guidelines’ recommendations. First, multiple factor analysis (MFA) was performed to investigate whether countries recommending the societal perspective as a base case (or as a complementary analysis) also exhibited similar positions regarding the inclusion, measurement, and valuation of societal cost. MFA is a multivariate method used to examine patterns of association among several groups of categorical dependent variables and identify clusters of observations with similar profiles. It constructs latent dimensions that represent the database information. Each axis is a synthetic construct that combines information from all variable groups, allowing the most salient patterns and relationships among observations to be visualized and interpreted. Distances between individuals have been computed using a weighted Euclidean distance, where each group of variables is normalized and scaled so that all groups contribute equally to the overall distance [37].

Secondly, cluster analyses were conducted to explore the potential existence of groups of countries sharing similar methodological recommendations regarding specific societal costs. The clustering procedure relied on an unsupervised machine-learning approach in which similarity between observations was defined using Euclidean distance. Countries were subsequently grouped according to these similarities, and the resulting clusters were visualized using a dendrogram to illustrate the hierarchical structure of the grouping. The final number of clusters was determined using multiple validation criteria: the average silhouette width method (with the optimal number of clusters corresponding to the highest average silhouette value (see Figure S3—Supplementary Materials)), the Partitioning Around Medoids (PAM), and two indicators, Kmeans withinness and Kmeans betweenness, to confirm the robustness of the selected clustering solution [38,39].

## 3. Results

### 3.1. Description of the HTA Bodies’ Guidelines

The methodological guidelines exhibited varying levels of detail regarding the measurement and valuation of societal costs. Table 2 summarizes the recommendations related to the societal perspective extracted from the nine HTA bodies’ guidelines.

Five of the nine HTA bodies recommended adopting a societal perspective as the base-case analysis (or as a co-base case in the case of the Institute for Clinical and Economic Review—ICER). Productivity losses were the societal cost most frequently cited in these recommendations, followed by informal care. However, substantial variation existed between countries regarding both the population affected by productivity losses and the types of productivity losses considered. Most guidelines focused on productivity losses borne by patients, whereas productivity losses incurred by caregivers were mentioned in only two guidelines. Absenteeism was referenced in six guidelines, presenteeism in five, and fewer than half addressed unpaid work—while leisure time was mentioned even less frequently.

For both productivity losses and informal care, the methodological guidance revealed marked heterogeneity across countries in terms of measurement and valuation approaches. Only one guideline specified a measurement method: The Netherlands recommended the use of the iMTA Productivity Cost Questionnaire (iPCQ) for capturing productivity losses. No other country provided specific tools or instruments to guide measurement. Similarly, with respect to informal care, only The Netherlands proposed a measurement method, recommending the use of a diary-based approach.

Among the three countries that made recommendations on the valuation of informal care (Canada, Spain, The Netherlands), the opportunity cost method was systematically mentioned. Concerning the valuation of productivity losses, guidelines more frequently referred to the friction cost method than to the human capital approach. The Netherlands, Canada and Germany explicitly recommended the exclusive use of the friction cost method, whereas France and Spain acknowledged both the human capital and friction cost methods without specifying a preferred approach. The glossary (Table S1, Supplementary Materials) provides a detailed discussion of the advantages and limitations of these methods.

Three countries (The Netherlands, the United States, and Canada) also referenced productivity losses related to patients’ education, although none provided recommendations on how such losses should be measured or valued. Most guidelines (six out of nine) mentioned the potential inclusion of unrelated future healthcare costs in economic evaluations. However, none offered methodological guidance on how these costs should be quantified or valued.

### 3.2. Comparison of Specific Recommendations on Societal Costs Given HTA Bodies’ Recommendation for Adopting a Societal Perspective as a Base Case

**Figure 1:**
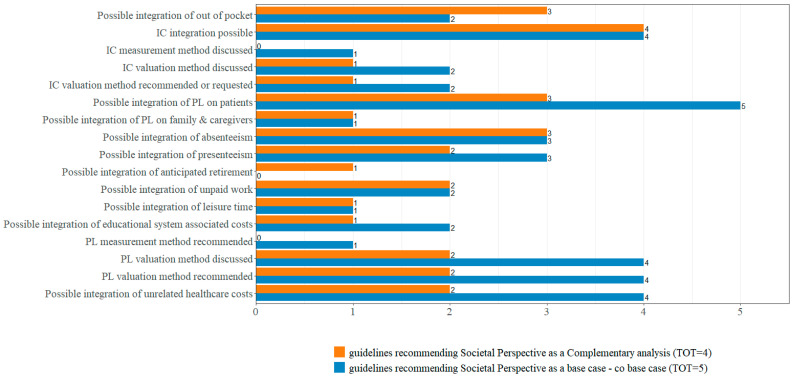
Distribution of methodological variables and recommendations for the inclusion of societal costs by type of perspective recommended by HTA bodies.

We observed a limited degree of homogeneity between the guidelines favoring the integration of societal costs as complementary analyses and those issued by HTA bodies recommending the societal perspective as a base case analysis (Figure 1). This figure only reports the items explicitly mentioned in the guidelines, and the types of costs that can be considered in the societal perspective for each HTA body. The results do not account for cross-country differences but distinguish guidelines recommending the societal perspective as a complementary analysis or as a base case. For example, among HTA bodies endorsing the societal perspective as a base case, only two addressed out-of-pocket and copayment costs, compared with three among those recommending the societal perspective as a complementary analysis. A similar pattern was observed for informal care: more HTA bodies referring to informal care recommended the societal perspective as a complementary analysis rather than as a base case. However, the methodological guidance for measuring and valuing informal care was generally more detailed among HTA bodies recommending the societal perspective as a base case. Regarding productivity losses, HTA bodies recommending the societal perspective as a base case referred more frequently to productivity losses affecting patients than those recommending it as a complementary analysis (five versus three). Nonetheless, apart from presenteeism, the types of productivity losses were more extensively developed in the guidelines recommending the societal perspective as a complementary analysis.

These findings were corroborated by the exploratory analyses using Multiple Factor Analysis (MFA). The MFA results showed that neither the recommendations on societal costs (Figure S1, Supplementary Materials) nor the macroeconomic variables (Figure S2, Supplementary Materials) were associated with whether HTA bodies recommended the societal perspective as a base case or as a complementary analysis. In both figures, after projecting the societal perspective as a supplementary variable, the two modalities (Base Case and Complementary) appeared close to the center of the factor map, indicating a weak association with the other variables included in the primary analysis. Taken together, the descriptive and exploratory analyses suggest that an HTA body’s position on adopting a societal perspective—whether as a base case or as a complementary analysis—cannot be interpreted as a proxy for its specific recommendations regarding societal costs or its macroeconomic context. Consequently, we proceeded with additional exploratory analyses to identify groups of countries based solely on their recommendations concerning the four categories of societal costs included in the extraction grid.

**Figure 2:**
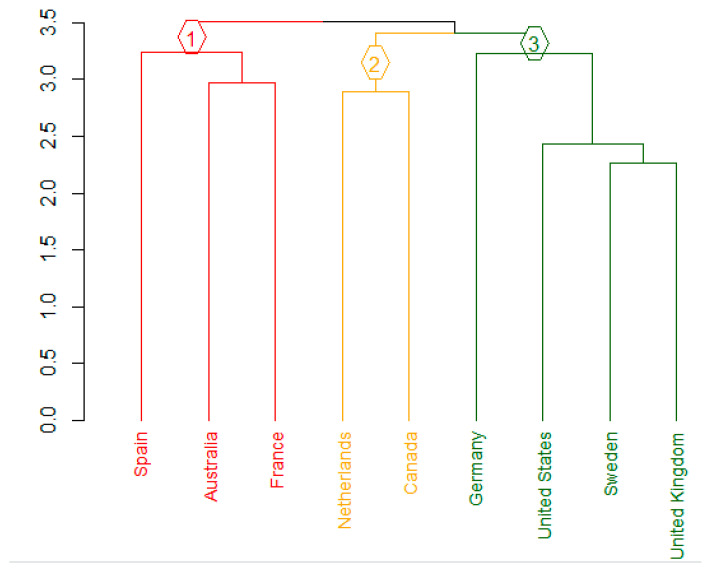
Cluster analysis—Dendrogram. Group ① (red): Spain, Australia, France; Group ② (yellow): The Netherlands, Canada; Group ③ (green): Germany, United States, Sweden, United Kingdom.

As displayed in the dendrogram (Figure 2), three distinct clusters of countries were identified (supported by the average silhouette width results; see Figure S3, Supplementary Materials):

Importantly, each of these clusters comprised a mix of countries recommending the societal perspective as a base-case analysis and those recommending it solely as a complementary analysis.

**Figure 3:**
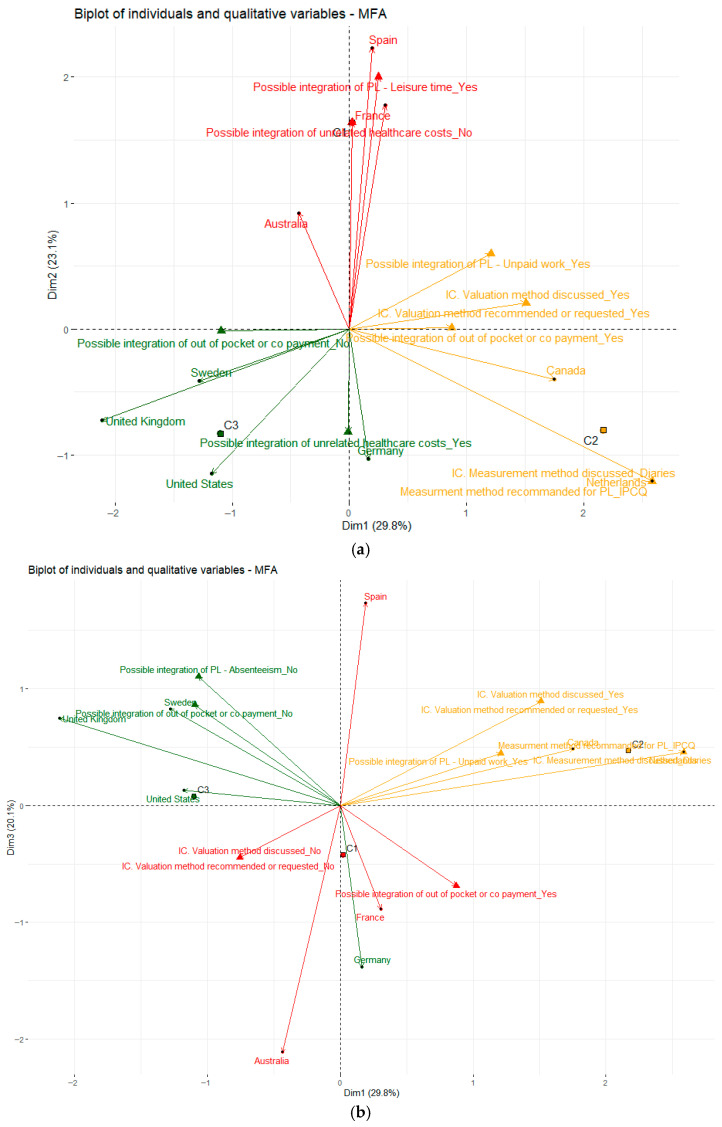
(**a**). Projected country groups on the top 10 modalities of societal cost recommendation variables: Axes 1–2. IC: informal care, IPCQ: IMTA productivity cost questionnaire, PL: productivity losses. (**b**). Projected country groups on the top 10 modalities of societal cost recommendation variables: Axes 1–3. IC: informal care, IPCQ: IMTA productivity cost questionnaire, PL: productivity losses.

The MFA performed on HTA bodies’ recommendations for the different groups of societal costs revealed a relative homogeneity in the distribution of modalities within the three domains—productivity losses, informal care, and out-of-pocket expenses. These groups of variables appeared correlated to varying degrees, suggesting the hypothesis that the presence of a positive (or negative) recommendation in one domain might be associated with similar patterns in the others. Based on this working assumption, we selected the top 10 variables contributing most strongly to the MFA axes and projected the three country clusters onto them (Figure 3a,b). This approach was intended to explore whether clusters exhibited internally consistent combinations of variables—thereby helping to characterize clusters through specific societal-cost recommendations—while also allowing us to identify areas where substantial heterogeneity persisted across clusters.

The multiple factor analysis (Figure 3a) shows that the three axes reflect different patterns in how HTA bodies address societal costs. Axis 1, which captures the largest share of variance, is predominantly driven by the variable *“use of the diary method to measure informal care”*, the most contributive modality on this axis. Countries in Cluster 2 (The Netherlands and Canada: Yellow) load strongly on this dimension, indicating greater methodological clarity regarding the valuation of informal care. Axis 2 is mainly shaped by the modality *“possibility to include leisure time in the societal perspective”*, a feature that characterizes Cluster 1 (France and Spain: Red), suggesting some convergence in how these countries’ HTA bodies approach this specific component of indirect costs. In contrast, Axis 3 (Figure 3b) is largely determined by the modality *“no possible integration of absenteeism”*, which is most strongly associated with Cluster 3 (Sweden and the UK: Green). While certain clusters show internal coherence around specific methodological choices (e.g., diary method, leisure time, or productivity losses), the MFA also highlights substantial heterogeneity both within and across clusters for other modalities related to societal costs. This points to a generally fragmented international landscape with only partial alignment on how societal elements should be integrated into health economic evaluations.

## 4. Discussion

This study aimed to analyze HTA bodies’ recommendations regarding the application of societal perspectives in health-economic assessment, using the most recent published guidelines issued by nine HTA bodies representing major pharmaceutical markets with established HTA systems. Beyond the descriptive assessment, we also sought to identify clusters of countries sharing relatively homogeneous positions on this topic. In line with previous work [26,40], we found that although most HTA bodies acknowledge the societal perspective, recommendations on whether it should be used as the base case or solely as a complementary analysis remain heterogeneous, with five countries endorsing its use as a base-case analysis, and four restricting its role to complementary analyses. Although the practical integration of the societal perspective in submitted dossiers has not been studied, the guidelines clearly highlighted cross-country interest in broadening the analysis of a health product’s efficiency to capture all relevant costs and externalities in the decision-making process. Our work highlights the uneven level of detail provided in the guidelines concerning the integration of societal costs. The most frequently discussed categories were out-of-pocket costs, productivity losses, and informal care—the latter two being defined as “common but inconsistently used elements of value” in the 2018 ISPOR “Value Flower” [32] and also corresponding to societal costs items most widely studied in the scientific literature on societal perspective in HTA [9,29,30,41,42]. Most guidelines described the types of direct non-medical costs to be included in health economic assessment, and generally recommended considering out-of-pocket costs, although their valuation often depends on national coverage policies.

Integrating informal care as a cost avoided or associated with a health technology is particularly challenging, as approximately 80% of long-term care in Europe is provided by informal caregivers [43]. In our review, although productivity losses for patients appeared in eight of the nine methodological guidelines, far fewer guidelines mentioned productivity losses for caregivers, unpaid work, leisure time, or anticipated retirement. This aligns with findings from the *Impact HTA* 2020 review [9]. Measuring informal care remains difficult, given that caregivers may not self-identify and that distinguishing “extra” care from everyday household support is inherently complex. Cultural norms and expectations regarding caregiving also influence definitions and reporting [44]. However, a substantial body of methodological literature discusses advantages and limitations of informal care measurement and valuation approaches [30,41,42,45,46,47]. To our knowledge, only The Netherlands has addressed several of the methodological challenges identified by Urwin et al. [30] and recommends a specific measurement tool for informal care (iPCQ).

Whereas leisure time was identified by Drummond et al. as a relevant cost component for health-economic evaluation [3], the scientific literature continues to show limited interest in valuing unpaid work and leisure time, both of which remain contentious [29,48]. As for informal care, HTA bodies could rely on a considerable methodological foundation to measure productivity losses. The recent review by Hubens et al. identified 42 instruments for measuring productivity losses—including absenteeism, presenteeism, and unpaid work [29]. While most of these tools were designed for patient-reported data collection and may not be fully adapted for societal-perspective analyses, Hubens et al. advocated the use of the iPCQ for estimating productivity losses across these domains [29].

With respect to valuation methods for productivity losses, the friction cost method—the most conservative and methodologically stringent—was mentioned more frequently than the human capital approach. However, applying the friction cost method in practice requires detailed knowledge of national labor market characteristics and the friction period. This period was estimated at 82 and 85 days in Germany and The Netherlands, respectively, whereas such estimates were not easily identifiable in the other countries included in our analysis, limiting the feasibility of this method elsewhere. For valuing informal care, the opportunity cost method was more frequently cited than the proxy good method. These methods, based on revealed preference methods, are relatively straightforward to implement compared with declared preference methods. The glossary (Table S1, Supplementary Materials) provides an overview of the advantages and limitations associated with each measurement and valuation method discussed.

It might have been expected that countries recommending the societal perspective as a base case would provide methodological guidance on measurement and valuation approaches to limit uncertainty and improve comparability across interventions with similar societal cost implications, as has The Netherlands. However, a key finding of our study is that the recommendation to adopt the societal perspective as a base-case versus a complementary analysis did not align with the level of methodological detail provided in the guidelines on the inclusion of societal costs. Overall, societal perspective recommendations appeared heterogeneous and were not determined by the presence or absence of detailed guidance on informal care, productivity losses, out-of-pocket costs, or other societal cost components. The three clusters of countries identified grouped together systems that did and did not recommend adopting the societal perspective as a base-case analysis.

This work has several limitations. First, it is based on a non-exhaustive review of guidelines from nine HTA bodies in developed countries, which may limit the external validity of the findings—particularly for emerging countries whose HTA systems are not yet fully established and where social policies may differ substantially. In addition, because the review was restricted to documents available in English, French, or Spanish, some international guidelines could not be considered, which may affect its generalizability.

Even if only the most recent guidelines available in each country were included, their publication dates ranged from 2003 to 2022, which can limit the comparability of data and the ability to extrapolate findings to the present date. Furthermore, although multiple experts were consulted to share their expertise on societal costs, refine the methodology used and validate the results, their voluntary participation may have introduced selection bias, potentially influencing the interpretation of the findings.

Secondly, the small number of countries included constrained the analytical methods. Given the limited sample size, we deliberately restricted our work to exploratory analyses, as formal comparative statistics would have been underpowered and potentially misleading. In these exploratory analyses, the first two to three components explained approximately 70% of the variance, which is consistent with results typically observed in small-sample studies [46,47]. These analyses confirmed that the information was adequately summarized along these axes, allowing meaningful visual projection of country profiles. Nevertheless, such results should be interpreted as preliminary and hypothesis-generating rather than confirmatory.

Another limitation pertains to the scope of societal costs considered. Except for productivity losses, several non-healthcare sectors defined in the ISPOR Second Panel’s impact inventory template (e.g., future unrelated consumption, criminal justice, education, housing, environment) were likely under-represented relative to their potential societal impact [32]. In particular, the environmental consequences of health interventions—such as resource use, water consumption, greenhouse gas emissions, the carbon footprint associated with transporting technologies, toxicity, waste management, and inefficiencies due to suboptimal use or recycling constraints—were only marginally addressed in the guidelines reviewed and remain sparsely explored in the literature [49,50] despite being highlighted as an emerging area of interest in the *2015 Health Technology Expert Review Panel Deliberative Framework* (CADTH) [51]. Moreover, the definitions of key concepts studied were not identical across countries, and the level of detail available varied (e.g., some definitions specified the use of time and costs to value informal care, whereas others did not detail this element), which can limit comparability across guidelines, but the definitions remained very similar (e.g., social services costs considered as indirect costs) and the impact on the results was thus deemed limited.

Finally, we found that similar macroeconomic characteristics—such as Gini index, the share of current health expenditure in GDP, out-of-pocket expenditure as a percentage of current health expenditure, GDP per capita, unemployment rate, or whether HTA recommendations are binding—are not associated with the countries’ acceptance of the societal perspective as a base case or complementary analysis. Nevertheless, other contextual factors—such as legislative constraints, the remit of national HTA bodies, cultural norms surrounding informal care, or structural differences in labor markets—may contribute to this heterogeneity. These factors warrant further research to provide a more comprehensive interpretation.

Despite these limitations, comparing data across nine HTA bodies enabled us to shed light on the debate regarding the integration of societal costs in health-economic evaluations worldwide. The choice to adopt the societal perspective varies considerably depending on national cultural norms and social policies. Nonetheless, the integration of societal perspectives in evaluations remains of the utmost importance while assessing the efficiency of a health product nowadays, as the optimal resource allocation cannot be reached without considering the full scope of both direct and indirect costs. This broader perspective is especially relevant for rare or chronic diseases, an area in which HTA bodies are increasingly conducting health-economic evaluations, and where the burden on family life and professional activity is substantial. It is also becoming increasingly important in relation to the environmental impact of health technologies, which can vary considerably depending on treatment characteristics (e.g., galenic form, number of injections). Ultimately, adopting a societal perspective can alter the estimated ICER—increasing or decreasing it relative to a narrower perspective—thereby potentially influencing access to health technologies.

## 5. Conclusions

Many HTA bodies currently do not recommend using a societal perspective as a reference analysis probably because societal costs fall outside the formal remit or mandate of these HTA bodies. Another contributing factor is the absence of clear and precise methodological guidance on how such costs should be measured and valued, which generates considerable uncertainty when attempting to incorporate them into cost-effectiveness analyses.

HTA bodies could draw inspiration from The Netherlands’ NHI guidelines and cost database, and more explicitly address the methodological challenges associated with both the measurement and valuation of societal costs. Developing a coherent “societal perspective doctrine” would require systematically identifying the expenditures with the greatest societal impact and defining appropriate estimation methods. Such a doctrine could include, for example, the development of minimum core sets of societal costs to be included in the societal perspective, along with a standardized costing guide specifying recommended measurement instruments (e.g., the widely used iPCQ questionnaire for productivity losses and diary methods for informal care). HTA bodies should also be encouraged to involve patients and caregivers in defining which societal costs are relevant to capture in economic evaluations. Indeed, NHI’s methodology is based on feedback from potential users of the national database (collected through an online survey), complemented by regular consultations with a panel of HTA-specialized health economists.

Building on this model, and in line with the recent proposal by Harvard et al. advocating for patient and public involvement in health economics modeling [52], HTA bodies should integrate patients and their significant others into the iterative process of validating and refining such a societal-cost doctrine. This approach would strengthen methodological robustness, improve legitimacy, and ensure that the societal perspective reflects the lived experiences of those most directly affected by disease and its broader consequences.

## Figures and Tables

**Table 1:** HTA bodies per country.

Country	HTA Body	Year
Australia	Pharmaceutical Benefits Advisory Committee (PBAC)	2016
USA	Institute for Clinical and Economic Review (ICER): Independent scientific institute; examines benefits and harms of medical interventions for patients	2020
The Netherlands	National HealthCare Institute (NHI)	2016
Canada	Canada’s Drug and Health Technology Agency (CADTH)	2017
France	National Health Authority (HAS)	2020
Germany	The Institute for Quality and Efficiency in Health Care (Institut für Qualität und Wirtschaftlichkeit im Gesundheitswesen, IQWiG): Independent scientific institute; examines benefits and harms of medical interventions for patients	2022
Sweden	Dental and Pharmaceutical Benefits Agency (Tandvårds- & läkemedelsförmånsverket, TLV)	2003
Spain	Health Minister of Health (Ministerio de Sanitad Servicios Sociales e Igualdad, MSSSI)	2010
United Kingdom	The National Institute for Health and Care Excellence (NICE)	2022

**Table 2:** Description of the guidelines about recommendations on societal costs.

	The Netherlands 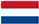	United States 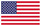	Germany 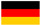	Spain 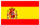	Sweden 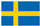	United Kingdom 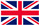	Canada 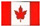	Australia 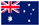	France 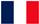	Total Number of Yes
Societal perspective recommended as a base case/co-base case										5
Possible integration of out-of-pocket costs										5
Possible integration of informal care										8
*1*. *Measurement method discussed*	Diary									1
*2*. *Valuation method discussed*										3
*3*. *Valuation method recommended*	OC			OC and PG and DP			OC and PG			3
**Possible integration of productivity losses (PL)**										
*1*. *PL on patients*										8
*2*. *PL on family and caregivers*										2
*3*. *Absenteeism*										6
*4*. *Presenteeism*										5
*5*. *Anticipate retirement*										1
*6*. *Unpaid work*										4
*7*. *Leisure time*										2
*8*. *Measurement method recommended*	IPCQ									1
*9*. *Valuation method discussed*										6
*10*. *Valuation method recommended*	FCM		FCM	HCM and FCM	HCM		FCM		HCM and FCM	6
Possible integration of educational system costs										3
Possible integration of environmental costs										0
Possible integration of unrelated healthcare costs										6

DP: declared preference, IPCQ: IMTA productivity cost questionnaire, FCM: friction cost method, HCM: human capital method, OC: Opportunity costs, PG: proxy good, PL: productivity losses.

## Data Availability

The original contributions presented in this study are included in the article/Supplementary Materials. Further inquiries can be directed to the corresponding author.

## Supplementary Materials

The following supporting information can be downloaded at: https://www.mdpi.com/article/10.3390/jmahp14010010/s1, Table S1: Detailed glossary; Table S2: Interview grid (The Netherlands case); Figure S1: Multiple factor analysis—Comparison of societal perspective acceptability and societal costs recommendations; Figure S2: Multiple factor analysis—Comparison of societal perspective acceptability and macroeconomics’ variables; Figure S3: Cluster analysis—Average silhouette width method; Figure S4: Multiple factor analysis—characterization of HTA bodies recommendations in terms of societal costs; Figure S5: Description of the steps conducted during this project.

## Abbreviations

The following abbreviations are used in this manuscript:

HTA	Health Technology Assessment
SHEER	Spillovers in Health Economic Evaluation and Research
SMA	Spinal Muscular Atrophy
NICE	United Kingdom’s National Institute of Health and Care Excellence
CADTH	Canada’s Drug and Health Technology Agency
NHI	National Health Institute
SBU	Swedish Agency for Health Technology Assessment
HAS	Haute Autorité de Santé
HRQoL	Health-Related Quality of Life
CUA	Cost–Utility Analysis
UK	United Kingdom
MFA	Multiple Factor Analysis
PAM	Partitioning Around Medoids
ICER	Institute for Clinical and Economic Review
iPCQ	iMTA Productivity Cost Questionnaire
SF-HLQ	Short Form-Health and Labour Questionnaire
HCM	Human Capital Method
HLQ	Health and Labour Questionnaire
USA	United States of America
PRODISQ	PROductivity and DISease Questionnaire
