# Supplementary material for: Adopting a Societal Perspective in Health-Economic Evaluation: Analysis of Nine HTA Methodological Guidelines on How to Integrate Societal Costs
Source: J Mark Access Health Policy. 2026 Feb 10;14(1):10. doi: 10.3390/jmahp14010010 (PMC12921813; doi:10.3390/jmahp14010010)

SUPPLEMENTARY MATERIALS

Table S1. Detailed glossary

**Societal perspective**	Perspective adopted for economic evaluation. Includes all relevant costs directly and indirectly related to a condition and/or intervention regardless of who bears the costs. These costs may be borne by the patient, his or her family or other sectors of society (education, justice, work, environment, etc.)
**Theoretical acceptability of the societal perspective by HTA agencies (« assessment »)**	The societal perspective is recommended for the baseline analysis, as a "co-benchmark analysis" or accepted as a complementary analysis for economic assessment
**Practical acceptability of the societal perspective by HTA agencies (« appraisal »)**	The societal perspective is actually used for health economic appraisal
**Indirect costs**	Resources lost indirectly as a result of illness or treatment. We can speak of externalities (positive or negative) on:<br>- Labor productivity (paid) lost by patients,<br>- Labor productivity (paid) lost by caregivers (e.g., partner/spouse, family and friends),<br>- Domestic (unpaid) productivity lost by patients (also referred to as non-work or unpaid productivity),<br>- Home (unpaid) productivity lost by caregivers,<br>- Leisure time lost per patient,<br>- Leisure time lost by caregivers
**Informal care**	A heterogeneous service, composed of various specific assistance tasks, either to cover the needs related to basic activities of daily living (eating, drinking, grooming, dressing, ...) or instrumental activities (meal preparation, shopping, visiting a health professional, chores) of a person with limited autonomy. In general, informal care is provided by one or more members of the social/emotional environment of the person who needs the care, mainly the couple or immediate family members.
**Unrelated future costs**	Morbidity-related costs, indirectly related to a disease or intervention, e.g.:<br>- permanent loss of productivity caused by premature death,<br>- medical costs that may occur during the life years gained (consequences of the life extension effect of an intervention)<br>Close to intangible costs ?
**Valuation methods : revealed preferences**	Set of methods used to value a non-market good/service that use real-life decision data to estimate the value of the non-market good/service: for example, for informal help, preferences are drawn from the decisions of informal caregivers or close substitutes (professional caregivers) in the labor market. Includes human capital and friction cost (paid time) and opportunity cost and replacement cost (unpaid time) methods. This set of methods includes human capital, friction cost and opportunity cost methods<br>**Advantages:** inexpensive to implement (only aggregate information on the hourly wage rate is needed, for example) - the friction cost method, however, implies knowing the duration of the "friction period" which is complex to estimate

	**Disadvantages:** do not take into account individual preferences concerning the non-market good/service (utility)
**Valuation methods : declared preferences**	A set of methods used to value a non-market good/service, using the preferences of respondents of interest (the patient/caregiver) for non-market goods (informal help, quality of life, etc.). For example, for informal care this method estimates the financial compensation that caregivers would be willing to accept to provide an extra hour of care for their sick relative. Includes any method that directly or indirectly asks an individual to state the willingness to pay/accept. This set of methods includes contingent valuation and conjoint analysis.<br>**Advantages:** take into account individual preferences (respondents, if they are rational, when they declare the value they give to their time, express through this value the level of utility they associate with it)<br>**Disadvantages:** costly to set up (need to set up and administer questionnaires) + non-response rate often high because the question of willingness to pay for a non-market good/service can be misunderstood because it is often associated with a completely fictitious scenario. The question on DAP may also shock the respondents ("how much would you be willing to pay to help your sick relative less?"), leading to a so-called "protest" non-response that should be treated correctly (with an adapted statistical model) before aggregating them.
**Valuation method : Human capital**	A method of valuing the paid time of the person affected by the illness or injury in question (patient). Originates in human capital theories. According to this theory, an increase in the stock of knowledge or human capital of an individual increases his or her productivity in the labor market, from which he or she derives monetary income, and in the non-market or domestic sphere, where he or she produces goods that enter into his or her utility function. To realize their potential productivity gains, individuals have an incentive to invest in education, job training and health. The labor productivity lost by patients, i.e. the opportunity cost of not working, can be approximated by the patient's remuneration in the labor market (the hourly/monthly/yearly wage...)
**Valuation method : Friction costs**	The main assumption of this approach is that the human capital method overestimates productivity losses by not considering the possibility of compensating for lost work time in situations of short-term work absences (either by the sick person him/herself when returning to work, or by his/her colleagues during his/her absence), or replacement in situations of long or permanent absence (after the so-called "friction period"). - For short-term absences of a worker due to illness, the effective working time will be reduced less than proportionally (about 80%). - For long term absences, a worker who is forced to leave his job permanently due to illness does not cause any loss of productivity for the company, since he will be replaced by another worker. The only loss occurs during the period when the position remains vacant (the friction period). The calculation of this friction period is crucial in this approach. The longer and more complex it is, the closer the results estimated by this approach will be to those of the human capital method. On the other hand, the shorter the friction period, the greater the differences will be.<br>**Advantages:** More conservative method (advantage for the payer), closer to reality concerning the hypothesis of non-full employment<br>**Disadvantages:** Poses the ethical problem of valuing the time of the inactive: moves away from neo-classical economic theory (value of work time = value of leisure time). Complex to implement because it requires information on the friction period and the replacement rate (ideally by socio-professional category)
**Valuation method : opportunity cost**	Method of valuing the unpaid time of the person affected by the disease or injury under consideration (patient/caregiver): consists in replacing the value of a non-market good/service by the value of his/her time on the labor market (value of the time of a median individual or an individual with similar socio-economic characteristics if the individual does not work).

	**Advantages:** consistent with neoclassical economic theory, only requires collecting the number of hours of informal/lost help by the patient (simple questionnaire, inexpensive to implement).<br>**Disadvantages:** does not take into account individual preferences, does not take into account the heterogeneity of helping tasks/type of unpaid time, may pose the ethical problem of valuing the time of the inactive
**Valuation method : replacement cost**	Method of valuing the unpaid time of the person affected by the illness or injury under consideration (patient/caregiver): Consists in replacing the value of a non-market good/service by the value of its closest market substitute (e.g., to approximate the hourly value of informal help we will use the hourly wage rate of a professional caregiver)<br>**Advantages:** allows to take into account the heterogeneity of the different unpaid tasks + does not generate "ethical" problems related to the valuation of the time of inactive people.<br>**Disadvantages:** does not take into account individual preferences, more time-consuming than the opportunity cost method, requires the elaboration of a more complex questionnaire - by type of task (risk of non-response).
**Presenteeism**	Time during which, without being absent from work, the worker's productivity is reduced to a greater or lesser extent, in relation to his or her health condition
**Unpaid work**	Can be divided into two categories:<br>- Unpaid productive time, i.e., time spent on unpaid work, such as household chores and voluntary work. Could be realized by a third-person.<br>- Leisure time : consists of “nonproductive” time that can be spent to one’s liking, including hobbies, sport activities, and lunch breaks.
**Out-of-pocket**	Amounts to be paid by patients or caregivers associated with the direct costs, not reimbursed by social security or mutual insurance, of patient care (hospital stays, transport and medication)
**Financial hardship**	Out-of-pocket expenses increased by the indirect costs of care (e.g., reductions in income associated with the reduction in work time related to the disease/intervention)
**Measurement method : Recall method**	Retrospective questionnaire to measure informal help time. For example, the caregiver is asked to estimate the amount of time spent helping their loved one over the past three months. The caregiver is asked to average their daily/weekly help time for different types of tasks.<br>**Advantages:** less time consuming for the respondent than the diary<br>**Disadvantages:** loss of information, approximations
**Measurement method : Diary**	"Diary" in which the caregivers will indicate each day (in real time) the type of tasks performed, and the time spent on each of them.<br>**Advantages:** close to reality, makes it possible to dissociate the times really linked to the activity of assistance and those linked to the usual domestic production<br>**Disadvantages:** time consuming for the respondent, time consuming for the analyst
**Direct non-medical costs**	Direct non-medical costs refer to the expenses directly attributable to the illness or its management but not related to medical care or healthcare services themselves. They include all out-of-pocket costs incurred by patients, families, or caregivers as a direct consequence of the disease or its treatment, such as transportation to healthcare facilities, accommodation during treatment, special dietary requirements, childcare during medical visits, or the purchase of disease-related equipment and supplies not covered by the healthcare system. These costs are considered distinct from direct medical costs and are typically included within the broader framework of societal costs, as they reflect the financial burden borne outside the formal healthcare sector

Table S2. Interview grid (Netherlands case)

INTERNAL EXPERT INTERVIEW MINUTES - NEThERLANDS

**Face to face meeting:** ☐	**Videoconference meeting:** ☒

IQVIA Netherlands expert:

- xxx

IQVIA France participants:

- xxx

Date/timeslot: June 7st – 02:30 to 04:30 pm

OBJECTIVES

- To validate and complete information/data from literature.
- The questionnaire will be divided into 7 parts, starting with general questions on the characteristics of your assessment agency(ies). Next, there will be a section on the societal perspective in general,. Then we'll discuss direct non-medical costs and out-of-pocket costs, then the costs of informal care, productivity losses and, finally, unrelated future costs. For each of these last 5 parts of the interview, we'll try to validate or reinforce with you what we've extracted from the NHI official guidelines (2016) & manual of costing (2017)

REMINDER

We have sent you a glossary to ensure that the terms we use mean the same thing to everyone. We would like to remind you of some important elements of this glossary:

- when we talk about the HTA body, we are talking about the assessor, the body responsible for the assessment.
- when we talk about the payer, we are talking about the body responsible for setting prices, i.e. the body responsible for the appraisal in practice

It is possible that these bodies are one and the same, depending on the country.

GENERAL QUESTIONS

1.Q1. Are your HTA body's recommendations binding or not on the funding of healthcare technologies?

(for instance in England and Scotland, NICE and SMC have an advisory role and the local NHS must fund all positive HTA recommendations… this is not the case in France (the HAS recommendation only "weigh" on final reimbursement decisions but are not mandatory).

- ☐ Yes
- ☐ No

1.Q1.A. If no, does HTA body's recommendation influence Choix multiple

- ☐ The reimbursement process
- ☐ The price-volume negotiations
- ☐ Other……………………………………..

1.Q2. Are there several HTA bodies in your country?

- ☐ Yes
- ☐ No

1.Q2.A. If yes, are they regional agencies?

- ☐ Yes
- ☐ No

If yes, can you give us a list of these agencies?...

1.Q2.B.If yes, do they assess the same type of health technology ?

- ☐ Yes
- ☐ No

If no, which difference ?..

1.Q2.C.If yes, do they have different guidelines concerning the use of societal perspective?

- ☐ Yes
- ☐ No

1.Q2.D How much of them recommend to adopt a societal perspective for HTA assessment ? …….. %

1.Q3. According to your experience, which societal cost might have the greatest impact on the ICER ?

- ☐ Informal care
- ☐ Productivity losses
- ☐ Direct non medical costs
- ☐ Unrelated health care costs
- ☐ Other…………………….

SOCIETAL PERSPECTIVE

1. Assessment validation

2.Q1. Do you confirm that the latest official guidelines are those published in 2016? specific to the Netherlands case

- ☐ Yes
- ☐ No

2.Q1.A. If no, what are the latest official guidelines? ……………………………………..

2.Q2. Do you confirm that the the article by Kanters TA et al. (2017) sums up the Dutch costing manual correctly? specific to the Netherlands case

- ☐ Yes
- ☐ No

RESEARCH ARTICLE

Update of the Dutch manual for costing studies in health care

Tim A. Kanters[1]*, Clazien A. M. Bouwmans[1], Naomi van der Linden[2], Siok Swan Tan[1,3], Leona Hakkaart-van Roijen[1]

1 Institute for Medical Technology Assessment, Erasmus School of Health Policy & Management, Erasmus University Rotterdam, Rotterdam, the Netherlands, **2** Centre for Health Economics Research and Evaluation, University of Technology Sydney, Sydney, Australia, **3** Erasmus MC University Medical Center, department of Public Health, Rotterdam, the Netherlands

* kanters@eshpm.eur.nl

2.Q2.A.If no, where can we find the last update of the costing manual (English version only)? …………..

2.Q3. Do you confirm this article recommends using societal perspective as a base-case? specific to the Netherlands case

- ☐ Yes
- ☐ No
- ☐ If no, what are the requirements for using a societal perspective? …………………………..

2. ***Assessment - Additional info***

2.Q4. Can you explain us what are the main differences between societal perspective and the healthcare perspective? Multiple choice

- ☐ integration of informal care among the resources to be costed
- ☐ integration of patient/family's productivity loss among the resources to be costed
- ☐ integration of indirect costs (including among others patient/family's productivity loss)
- ☐ integration of unrelated health care costs among the resources to be costed
- ☐ Other: which resources? …………………………

2.Q5. Which payers are considered within the healthcare perspective?

whole public sector
public healthcare sector
private sector
other………………………

Q6. Is it compulsory to include perspective of the Healthcare System perspective in addition of societal perspective?

- ☐ Yes
- ☐ No

2.Q7. Does this perspective include social care ?

- ☐ Yes
- ☐ No

2.Q8. Does the societal perspective always have been the base-case perspective for your HTA body?

Yes
No

DIRECT NON-MEDICAL COSTS

1- Assessment validation

3.Q1. Do you confirm that the costs presented in the article summarizing the Dutch manual of costing are exhaustive?

Yes
No

Paramedical care (per visit)	
- Physical therapy	€ 33
- Exercise therapy	€ 34
- Speech therapy	€ 30
- Occupational therapy	€ 33
Elderly care	
- Inpatient elderly care incl. daycare, per day	€ 168
- Daycare	€ 67
Home care (per hour)	
- Household activities	€ 20
- Personal care at home	€ 50
- Support at home	€ 58
- Nursing at home	€ 73
- Home treatment	€ 120
Mental health care (per visit)	
- Primary care physician	€ 66
- Nurse practitioner	€ 17
- Social worker	€ 65
- Primary care psychologist	€ 64
- Independent psychotherapist	€ 94
- Independent psychiatrist	€ 94
- Ambulatory consultation general institution	€ 98
- Inpatient day	€ 302
- Daycare treatment	€ 169
Rehabilitation therapy	

Health care service	Reference price [a]
- Rehabilitation therapy per hour	€ 153
- Daycare treatment (children)	€ 521
- Daycare treatment (adults)	€ 460
Health care for disabled patients	
- Inpatient care for mentally disabled patients incl. daycare, per day	€ 209
- Inpatient care for physically disabled patients incl. daycare, per day	€ 205
- Inpatient care for aurally disabled patients incl. daycare, per day	€ 310
- Inpatient care for visually disabled patients incl. daycare, per day	€ 217

2- Assessment - Additional info

3.Q2. According to you, which direct non-medical costs requested by your HTA body are missing in our list?

……………………………………………………..

3.Q3. How are this type of resources covered? Choix multiple

- Social security
- Public financing
- Private aide
- Private insurer
- Out-of-pocket
- If so, what is the extent of the out-of-pocket for the patient? ………………………………………..

3.Q4. If a direct non-medical cost is publicly funded, is it recommended to include it in a health care system perspective?

Yes
No
- Yes, even if it is not publicly funded

3.Q5. Is there any recommendation about the measurement method for direct non-medical costs in your guidelines?

Yes
No
- If yes, which data source is recommended? ………………………..

3.Q6. Is there any recommendation about the valorization method for direct non-medical costs in your guidelines?

Yes
No
- If yes, which data source is recommended? ………………………..

OUT- of -POCKET

1. Assessment validation

4.Q1. Do you confirm the Dutch manual of costing does not mention out-of-pocket integration within economic assessment but do mention co-payments? specific to the Netherlands case

Yes
No
There are considered to be identical

4.Q1. A. If out-of-pocket is mentioned, please can you indicate us where we can find this information within your guidelines/Dutch manual of costing? …………………………

2. Assessment - Additional info

4.Q2. How do you define out-of-pocket?..

4.Q3. Is the integration of out-of-pocket costs recommended when adopting a societal perspective?

Yes
No

4.Q4. Can you indicate how the Dutch manual of costing recommends measuring these costs Choix multiple:

- Survey - Questionnaires (which ones?)
- Other ……………..

4.Q5. Can you indicate how the Dutch manual of costing recommends

to value these costs Choix multiple:

- official publications
- the accounts of centers
- market prices
- the rates applied to NHS service provision contracts
- national data

INFORMAL CARE

1. Assessment validation specific to the Netherlands case

5.Q1. Do you confirm that your guidelines recommend informal care cost integration within health economic assessment?

Yes
No

5.Q2. Do you confirm that your guidelines value informal care as a direct non-medical cost instead of an indirect cost?

Yes
No

5.Q3. Do you confirm that your guidelines propose to value informal care with the opportunity cost method?

- Yes
- No

5.Q3.A. If no, do your guidelines also recommend the use of replacement method to value informal care?

- Yes
- No
- Other method: …………………………………………………………………………………………

5.Q4. Do you confirm that the reference price for informal care, calculated with the opportunity cost method should mandatorily be used to value informal care Choix multiple?

- Yes
- No, it is not mandatory

Other method: …………………………………………………………………………………………

2. Assessment - Additional info

5.Q5. How informal care is defined in your guidelines? ……………………………………

5.Q6. Does informal care benefit from state aid or public/private insurance?

Yes
No

INDIRECT COSTS

1. Assessment validation specific to the Netherlands case

6.Q1. Do you confirm that your guidelines recommend patient's productivity losses integration within health economic assessment?

Yes
No

6.Q2. Do you confirm that your guidelines don't recommend caregiver's productivity losses integration within health economic assessment?

Yes
No

6.Q3. Do you confirm that your guidelines mention the use of the iPCQ questionnaire to measure productivity losses?

Yes
No

6.Q4. Do you confirm that productivity losses include:

- 6.Q4.A. Reduction in paid work (absenteeism and presenteeism)
  Yes
  No
- 6.Q4.B. Reduction in unpaid work
  Yes
  No

2. Assessment - Additional info

6.Q5. How is productivity loss covered in your country Choix multiple?

- collectively (social security, public financing, state aid)
- it is at the expense of the patients and/or their families

6.Q5. A. If so, what is the extent of the out-of-pocket for the patient?...

6.Q6. Even if it is not mentioned in your guidelines, do productivity losses, due to reduction in paid work, integrate:

- 6.Q6. A. Absenteeism
  Yes
  No

- 6.Q6. B. Presenteeism
  Yes
  No

- 6.Q6. C. Anticipate retirement
  Yes
  No

- 6.Q6. D. Part time work
  Yes
  No

6.Q7. In addition to paid work and unpaid work, does the valuation of productivity losses integrate leisure time lost?

- Yes
- No

6.Q7. A. If no, why?...

6.Q8. In addition to productivity losses, which indirect costs can be considered Choix multiple

- Educational system
- Judicial System

- Other, such as: ……………………………………

UNRELATED HEALTH CARE COSTS

1. Assessment validation specific to the Netherlands case

7.Q1. Do you confirm that your guidelines mention unrelated health care costs?

Yes
No

7.Q2. Do you confirm that unrelated health care costs should be calculated based on the average health care usage per person?

Yes
No

7.Q3. Do you confirm that the Dutch manual of costing mention the use of Practical Application to Include future Disease costs (PAID)?

Yes
No

2. Assessment - Additional info

7.Q4. Do they have to be integrated when adopting a societal perspective?

Yes
No

7.Q5. Can these costs be compared with intangible costs?

Yes
No

Figure S1. Multiple factor analysis – Comparison of societal perspective acceptability and societal costs recommendations

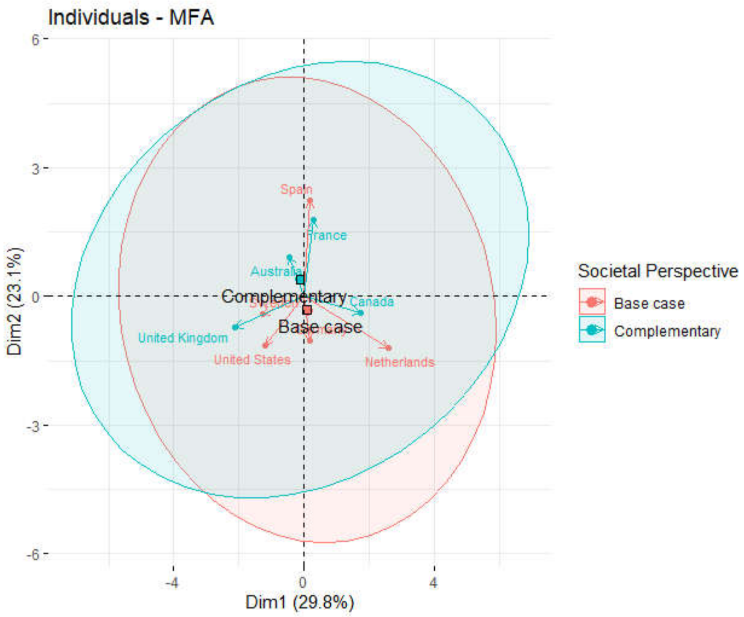

Multiple Factor Analysis (MFA) was performed on societal cost recommendation and societal perspective was added as supplementary variable. The acceptability of societal perspective as a base case/complementary analysis is unrelated to societal costs recommendations. Indeed, base case and complementary analysis were not well represented (close to the intersection of the axes 1 & 2) and countries were not homogenously grouped according to their societal perspective acceptance on axes 1 & 2 of the MFA (representing ~50% of the explained variance).

Figure S2. Multiple factor analysis – Comparison of societal perspective acceptability and macroeconomics' variables

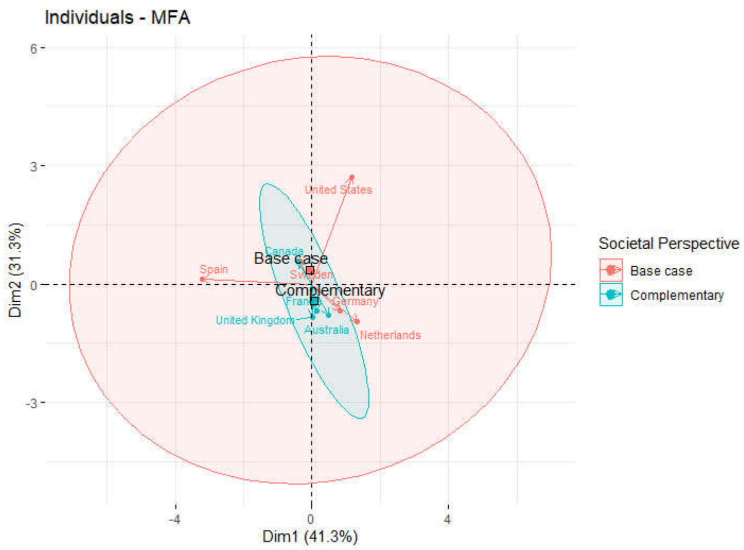

Multiple Factor Analysis (MFA) was performed on the following macroeconomic variables from the World Bank open data (https://data.worldbank.org/indicator/SH.XPD.CHEX.GD.ZS?end=2019&name_desc=false&start=2000) :

- 2019 Gini index
- 2019 current expenditure on health – share of Gross Domestic Product (GDP)
- 2019 Out-of-pocket expenditure as percentage of current health expenditure (%)
- 2019 GDP per capita, current prices (U.S. dollars per capita)
- 2019 Unemployment rate (Percent)

And from HTA country experts' interviews

- HTA body's recommendations binding or not on the funding of healthcare technologies

Societal perspective was added as a supplementary variable. Figure S2 shows that the acceptability of societal perspective as a base case/complementary analysis is unrelated to these macroeconomics variables. In other words, it is not because countries have close macroeconomic characteristics that they will accept the societal perspective as a base case or a complementary analysis homogeneously.

Figure S3. Cluster analysis – Average silhouette width method

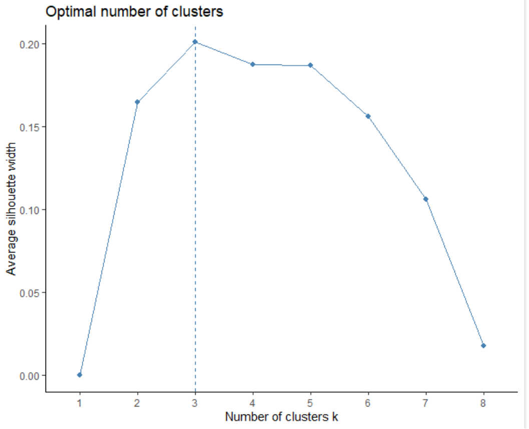

Figures S4 a & 4.b Multiple factor analysis – characterization of HTA bodies recommendations in terms of societal costs

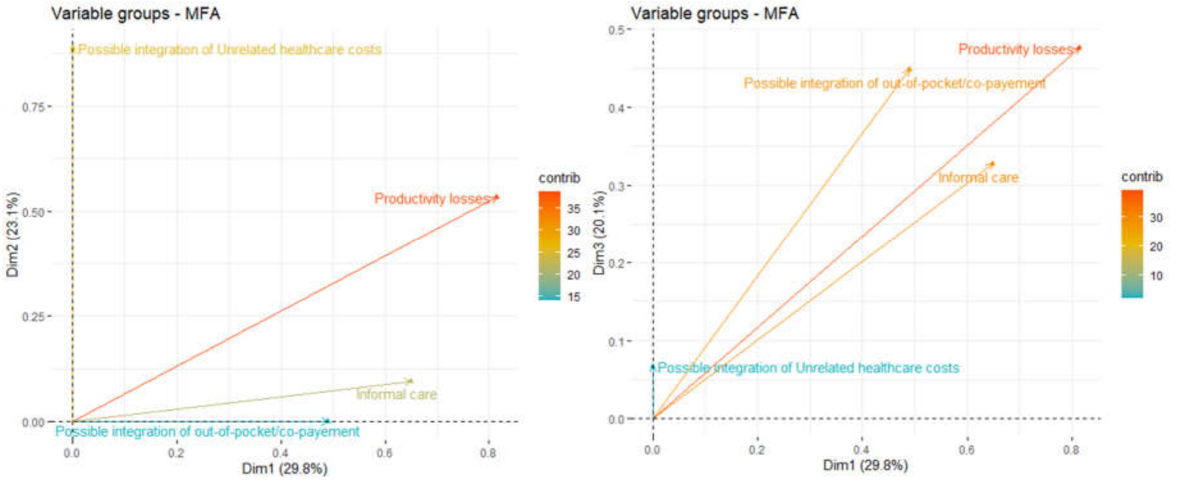

MFA was performed on HTA bodies recommendation and group of variables were represented on axes 1, 2 & 3, which contributed to more than 70% of the axis formation. **Figure S4.a** showed that “Informal care" group of variables and possible integration of out-of-pocket/co-payment variable were associated with axis 1 and that possible integration of unrelated health care costs was strongly associated to axis 2. As shown in **Figure S4.b**, axis 3 was also associated with informal care and possible integration of out-of-pocket/co-payment variable but in a lesser extent than axis 1. Axes 1 & 3 were both associated to productivity losses group of variables. Since the longer the arrow, the greater the weight of the variable in the analysis (i.e., it provides information and has discriminatory power), informal care (axis 1) and unrelated health care costs (axis 2) made important contribution to the definition of the global societal perspective recommendations (recommending societal perspective as a base case or as a complementary analysis). Contribution of both productivity losses & out-of pocket/copayment variable was more qualified.

Figure S5 Description of the steps conducted during this project

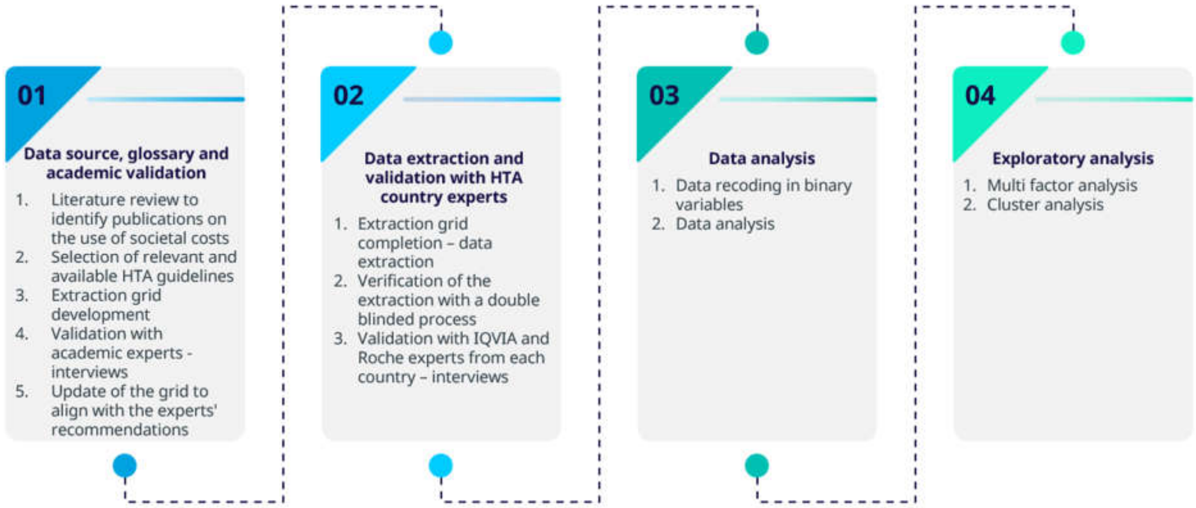

Supplement: Supplementary file 1 [file jmahp-14-00010-s001.zip › jmahp-4028569-supplementary.pdf]
